# CircRNAs: Roles in regulating head and neck squamous cell carcinoma

**DOI:** 10.3389/fonc.2022.1026073

**Published:** 2022-11-22

**Authors:** Xiao Han, Ruxian Tian, Cai Wang, Yumei Li, Xicheng Song

**Affiliations:** ^1^ Department of Otorhinolaryngology Head and Neck Surgery, Yantai Yuhuangding Hospital, Qingdao University, Yantai, Shandong, China; ^2^ Department of Otorhinolaryngology Head and Neck Surgery, Shandong Provincial Clinical Research Center for Otorhinolaryngologic Diseases, Yantai, Shandong, China; ^3^ School of Clinical Medicine, Weifang Medical University, Weifang, Shandong, China

**Keywords:** head and neck squamous cell carcinoma, miRNA sponge function, tumor regulation, tumor microenvironment, circular RNAs

## Abstract

Head and neck squamous cell carcinoma (HNSCC), the most common head and neck malignant tumor, with only monotherapy, is characterized by poor prognosis, and low 5-year survival rate. Due to the lack of therapeutic targets, the targeted drugs for HNSCC are rare. Therefore, exploring the regulation mechanism of HNSCC and identifying effective therapeutic targets will be beneficial to its treatment of. Circular RNA (CircRNA) is a class of RNA molecules with a circular structure, which is widely expressed in human body. CircRNAs regulate gene expression by exerting the function as a miRNA sponge, thereby mediating the occurrence and development of HNSCC cell proliferation, apoptosis, migration, invasion, and other processes. In addition, circRNAs are also involved in the regulation of tumor sensitivity to chemical drugs and other biological functions. In this review, we systematically listed the functions of circRNAs and explored the regulatory mechanisms of circRNAs in HNSCC from the aspects of tumor growth, cell death, angiogenesis, tumor invasion and metastasis, tumor stem cell regulation, tumor drug resistance, immune escape, and tumor microenvironment. It will assist us in discovering new diagnostic markers and therapeutic targets, while encourage new ideas for the diagnosis and treatment of HNSCC.

## Introduction

Head and neck squamous cell carcinoma (HNSCC), originating from the mucosal epithelium of the mouth, pharynx, and larynx, is the sixth most common cancer worldwide (1). Approximately 42% of the patients with HNSCC are diagnosed as intermediate or advanced disease, and these patients often have local lymph node metastases (2). Moreover, HNSCC has a low response to treatment and high drug resistance, with a 5-year survival rate of only 40-50% and a poor prognosis (3). Therefore, the early diagnosis, intervention and seeking potential therapeutic targets for HNSCC are of great significance to improve the prognosis and survival quality of patients. In recent years, an increasing number of studies have been devoted to the identification of specific biomarkers for the clinical decision-making. Currently, some emerging biomarkers have encouraged new ideas for the clinical diagnosis and treatment of HNSCC, including some non-coding RNAs (ncRNAs), like long non-coding RNAs (lncRNAs) and microRNAs (miRNAs), whose aberrant expression has been shown to be closely associated with the development of HNSCC (4). Particularly, circular RNAs (circRNAs), the newly discovered non-coding RNA molecules with closed-loop structure, have been proven to be a kind of new and stable biomarkers for diagnosis and treatment of HNSCC (5). CircRNAs exist widely in different species and cell lines, and are abundant, stable, conserved, and tissue-specific. In humans, they can be secreted into blood, saliva, and exosomes, playing an significant role in the tumor microenvironment. With the rapid development of next-generation sequencing technology, the basic structure and function of circRNAs have been fully confirmed, among which the functions ass HNSCC biomarkers and miRNA sponge, are extensively studied in recent years. However, the specific regulatory mechanism of circRNAs in the critical process of HNSCC development is still worth exploring. This review comprehensively described of the regulatory role of circRNAs in the development of HNSCC, interpreting the role and mechanism of circRNAs in the occurrence of this cancer, offering clues for diagnostic markers and therapeutic targets, and inspiring new ideas for the diagnosis and treatment of HNSCC.

## CircRNAs

**Figure 1:**
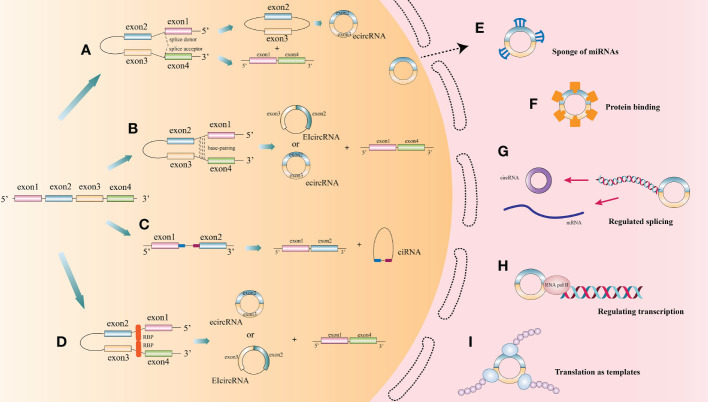
The biogenesis process and function of circRNA. **(A)** Lariat-driven circularization: During splicing of mRNA precursors, exon skipping produces a lariat intermediate containing both exons and introns. This intermediate will be reverse spliced and introns will be removed to form ecircRNAs. **(B)** Some introns on either side of one exon of a circRNA contain reverse complementary sequences. the RNA duplexes form two different types of circRNAs by variable splicing: circRNAs with introns and without introns, or introns within and on either side of one exon, which can compete for RNA pairing. **(C)** CiRNAs model. CiRNAs are generated by splicing reactions of introns. GU-rich elements near the 5’-splice site and C-rich elements near the branching site stabilize CiRNAs to avoid splicing. **(D)** RNA-binding proteins (RBPs) drive the cyclization of circRNAs. RBPs facilitate the formation of circRNAs by bridging the two sides of introns together. **(E)** CircRNAs act as endogenous competitive RNAs or miRNA sponge in cytoplasm. **(F)** CircRNAs act as protein sponge or protein storage library. **(G)** CircRNAs regulate splicing. **(H)** CircRNAs regulate transcription. **(I)** Translation function of circRNA.

CircRNAs, first identified in viroids in 1976, are classified as non-coding RNA (6). They are characterized by extensive distribution, stability and high conservation (7). In humans, circRNAs can express in the vast majority of tissues (8–12). However, different circRNAs express with tissue specificity or developmental stage specificity (13). Based on different splicing methods, circRNAs are divided into three main categories: exon circRNAs (ecircRNAs), exon-intron circRNAs (EIciRNAs), and intron circRNAs (ciRNAs). Many studies have focused on the most common circRNAs, namely ecircRNAs (9, 14). According to recent research, the cyclization modes of circRNAs can be divided into intron cyclization and exon cyclization. There are several explanation models for the cyclization mechanism of circRNA: lariat-driven circularization (9, 15–19) (**Figure 1A**), intron reverse complementary sequence driven cyclization (9, 20, 21) (**Figure 1B**), ciRNAs model (22) (**Figure 1C**), and RNA binding protein driven cyclization (23, 24) (**Figure 1D**).

At present, the functions of circRNAs as endogenous competitive RNAs or miRNA sponge in cytoplasm are wide studied (**Figure 1E**). Competitive endogenous RNAs (ceRNAs) contain shared miRNA response elements (MRE), such as mRNAs, pseudogenes, and LncRNAs), enabling them to competitively bind miRNAs. CircRNAs with the same specific miRNA binding site may regulate the activity of competing endogenous RNA, thereby interfering the effect of miRNA on its target through competitive miRNA binding (14). Cerebellar degeneration‐related protein 1 antisense RNA (CDR1as), also known as circular RNA sponge for miR-7 (ciRS-7), was reported to antagonize miR-7 availability (25). By targeting different miRNAs and mRNAs, circRNAs are involved in the pathogenesis of a variety of diseases, including neurological diseases (26), psychiatric diseases (27), autoimmune diseases (28), cardiovascular diseases (29, 30), cartilage degenerative diseases (31, 32), diabetes (33, 34), pulmonary fibrosis (35, 36), and various cancers (10, 37–39).

CircRNAs can also function as a protein sponge or protein storage library (**Figure 1F**). For example, circMBL can play a crucial role in balancing the expression levels of MBL mRNA and circMBL by isolating the excessive MBL protein (40).

CircRNAs regulate alternative splicing or transcription (**Figures 1G, H**). Back spliced of circRNAs may compete with linear spliced pre-mRNA for splicing sites. For example, the circMBL generated from the second exon of MBL has MBL binding sites flanking the intron (40). Thus, MBL level significantly affects circMBL biogenesis. Nuclear EIciRNAs with intronic sequences from parental genes, such as circEIF3J and circPAIP2, can interact with U1 small nuclear ribonucleotide proteins (snRNPs) and then bind to RNA polymerase II (Pol II) on the promoter of the parental gene, thereby enhancing gene expression (41). Besides, knocking down the expression of ElciRNAs has the potential to reduce the transcription of their parental genes (41).

Since the ring structure of circRNA is formed by attaching its 3’-end with 5’-end through a unique back splicing, reducing essential translation elements such as 5’-caps and poly-A tails, resulting in the blocked translation of most circRNAs. However, some circRNAs, such as circZNF609, circMb1, circ-FBXW7, circPINTexon2, and circ-SHPRH (42–47), exerted their regulatory functions as polypeptides translated by ribosomes at internal ribosome entry sites (IRESs) or by incorporating with m6A ribonucleic acid in the untranslated region (UTR) on the 5’-end (48–53), thus exerting their regulatory functions (22) (**Figure 1I**).

With the rapid development of bioinformatics, thousands of circRNAs in humans have been identified. CircRNAs have unique characteristics and a broad range of functions. As new important participant in the ncRNA network, circRNAs have been identified as key regulatory factors in various cancers. Based on existing knowledge of HNSCC characteristics, we assumed that circRNAs may play a possible role in various aspects of HNSCC.

### CircRNAs regulate tumor cell proliferation

Cancer cells can synthesize and respond to growth factors (GFs), which in turn promote their own proliferation, forming a positive feedback growth signaling circuit (54), that is, continuous proliferation signal stimulation and infinite division. This is the basis of tumorigenesis.

**Figure 2:**
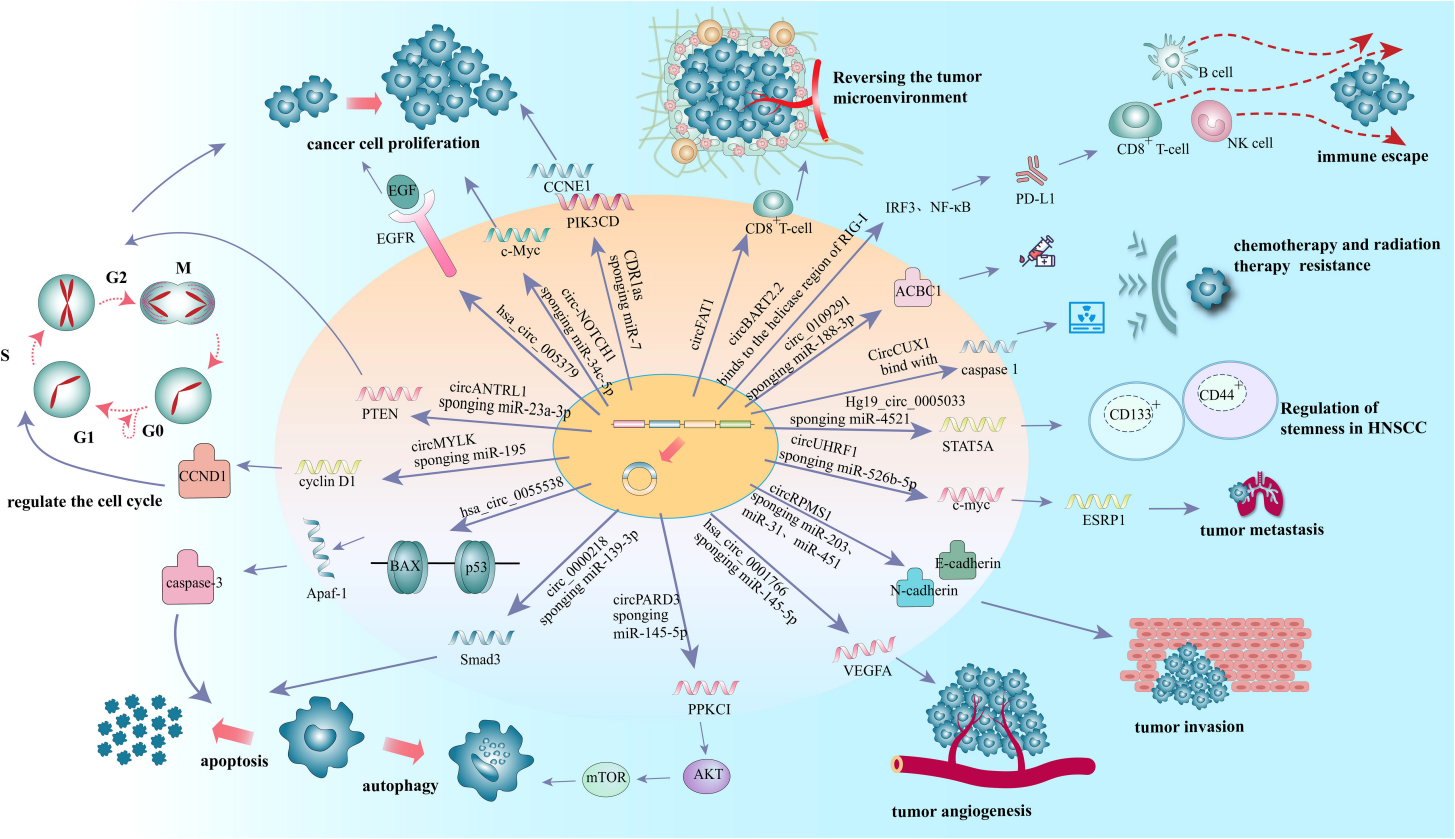
The regulatory roles of circRNAs in the occurrence and development of HNSCC.

More than 90% of HNSCC patients were tested positive for epidermal growth factor receptor (EGFR) (55), which is highly expressed in a variety of solid tumors and triggers an intracellular growth factor transduction cascade to regulate cell growth (56, 57). EGFR is overexpressed in HNSCC and can activate related pathways leading to the proliferation of cancer cells (58). Previous studies have confirmed that circRNAs can affect the proliferation of tumor cells by regulating the expression of EGFR in lung cancer (59), colorectal cancer (60), glioblastoma (61), melanoma (62) and other malignant tumors. In oral squamous cell carcinoma (OSCC), hsa_circ_0005379 is located in the upstream of EGFR. Overexpression of hsa_circ_0005379 expression can inhibit the expression levels of EGFR and phosphorylated EGFR, thus regulating the proliferation of OSCC tumor cells (63) (**Figure 2** and **Table 1**). Overexpression of the circZNF609 can stimulate miRNA-134-5P, and then up-regulate EGFR to promote the proliferation of laryngeal squamous cell carcinoma (LSCC) cells and the progression of LSCC (64) (**Table 1**).

**Table 1:** Mechanisms of action and biological functions of key circRNAs involved in HNSCC.

CircRNAs	Chromosome	Gene symbol	Splicing type	Cancer type	Expression	Targets/effectors	Biological function	Potential function	Gene structure	Reference
hsa_circ_0005379	chr10	GDI2	EIciRNAs	OSCC	down	EGFR	Oncogenic functions	Therapeutic target	–	(63, 176, 177)
circZNF609	chr15	ZNF609	EIciRNAs	LSCC	up	miRNA-134-5P /EGFR	Promotes proliferation	Therapeutic target	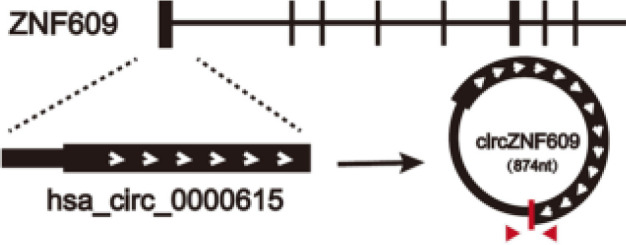	(64, 178)
circ-NOTCH1	chr9	NOTCH1	EIciRNAs	NPC	up	miR-34c-5p /c-Myc	Oncogenic functions	–	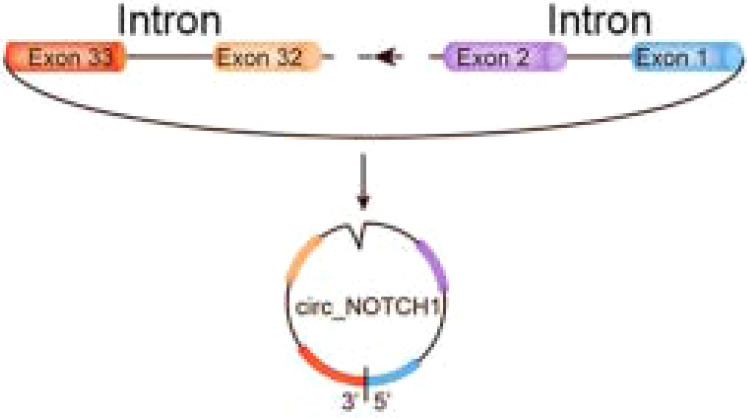	(179)
circCAMSAP1	chr9	CAMSAP1	EIciRNAs	NPC	up	SERPINH1 /c-Myc	Oncogenic functions	Therapeutic target	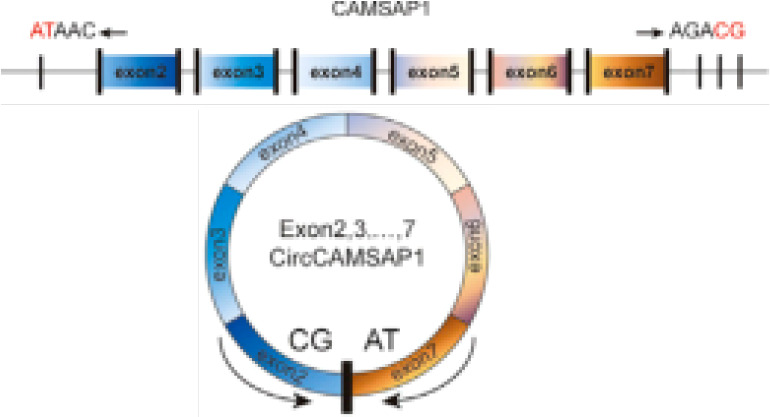	(67, 180)
circUHRF1	chr19	UHRF1	EIciRNAs	OSCC	up	miR-526b-5p /c-Myc	Promotes proliferation	Therapeutic target	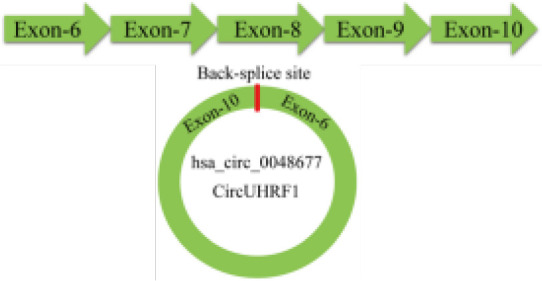	(68, 181)
circCDR1as	chrX	CDR1	EIciRNAs	LSCC	up	miR-7	Promotes proliferation	Prognosis and biomarker	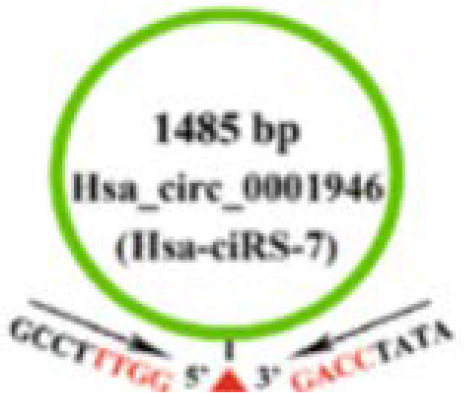	(25, 182)
				OSCC	up	miR-671-5p	Promoted autophagy	Therapeutic target	–	(183)
circANTRL1	chr10	ANTRL1	EIciRNAs	OSCC	down	miR-23a-3p /PTEN	Induce cell cycle arrest	Therapeutic target	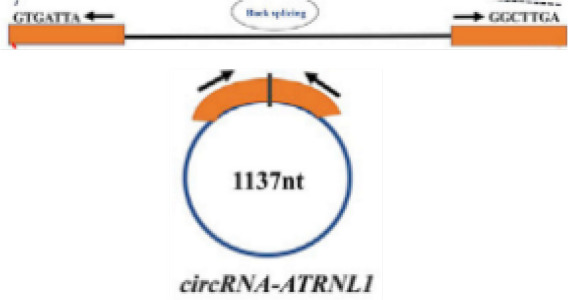	(72)
hsa_circ_0006232	chr6	TRERF1	EIciRNAs	LSCC	up	PTEN	Oncogenic functions	Therapeutic target	–	(73, 176, 177)
circMYLK	chr3	MYLK	EIciRNAs	LSCC	up	miR-195 /cyclin D1	Promotes proliferation accelerates cell cycle transition	Therapeutic target	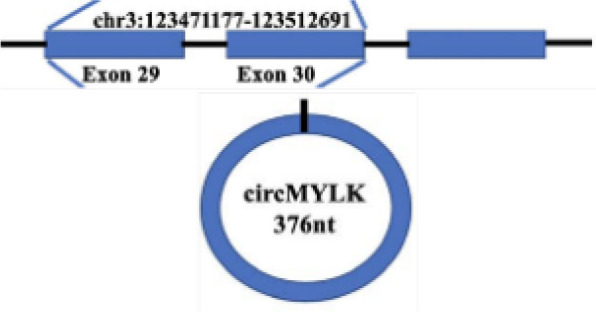	(74, 184)
circ-CCND1	chr11	CCND1	EIciRNAs	LSCC	up	miR-646/CCND1	Accelerates cell cycle transition	Therapeutic target	–	(75, 176, 177)
circ_0000745	chr17	SPECC1	EIciRNAs	OSCC	up	miR-488/CCND1	Induced cell cycle arrest	–	–	(76, 176, 177)
has_circ_0055538	chr2	RMND5A	EIciRNAs	OSCC	down	p53/Bcl-2 /Caspase	Tumor suppressor	Therapeutic target	–	(80)
circ_0005320	chr17	SEPT9	EIciRNAs	OSCC	up	miR-486-3p /miR-637	Oncogenic functions	Therapeutic target	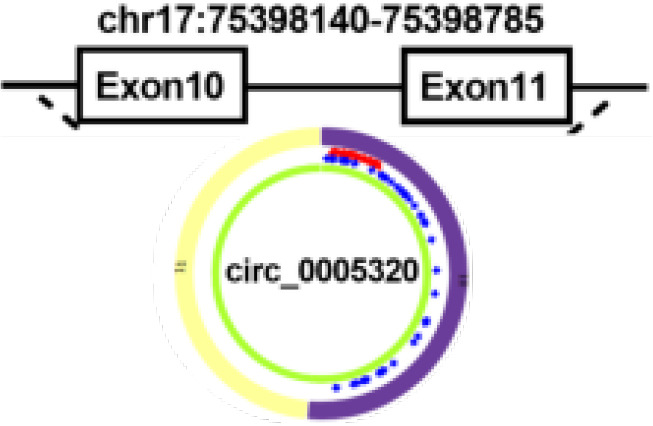	(81)
circ_0000218	–	–	–	LSCC	up	miR-139-3p /Smad3 axis	Oncogenic functions	Therapeutic target	–	(83)
circPARD3	chr10	PARD3	EIciRNAs	LSCC	up	PRKCI-Akt- mTOR	Inhibit autophagy	Therapeutic target and biomarker	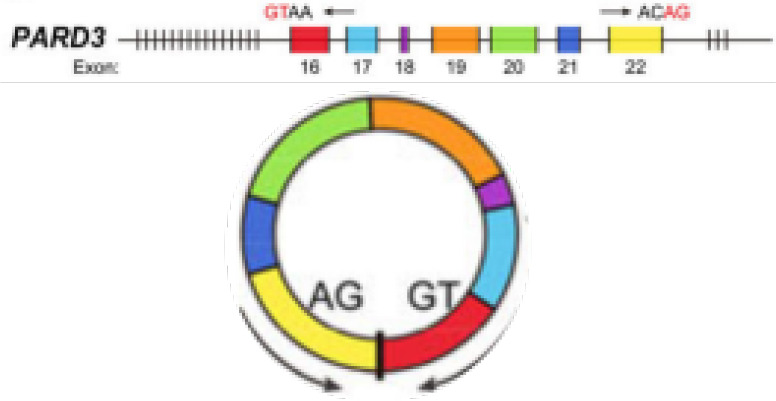	(93)
hsa_circ_0001766	chr7	PDIA4	EIciRNAs	OSCC	up	miR-877-3p /VEGFA	Oncogenic functions	Biomarker	–	(101, 176, 177)
circRPMS1	–	RPMS1	–	NPC	up	miR-203, miR-31, and miR-451	Promote EMT, Oncogenic functions	Therapeutic target	–	(106)
circEPSTI1	chr13	EPSTI1	EIciRNAs	OSCC	up	miR-942-5p	Promote EMT, Oncogenic functions	Therapeutic target	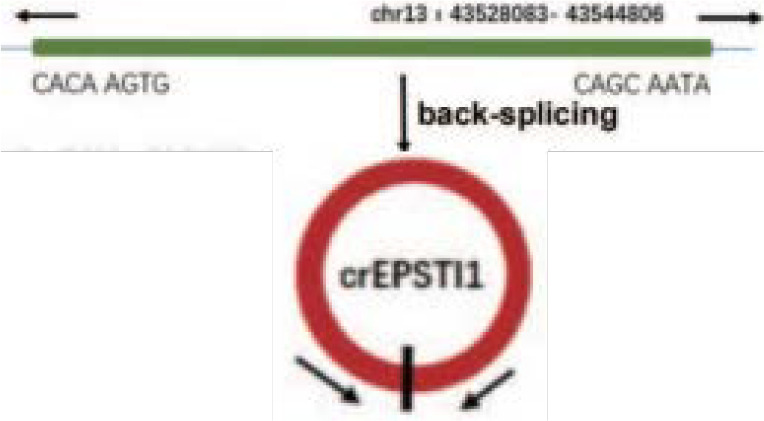	(107, 185)
circCORO1C	chr12	CORO1C	EIciRNAs	LSCC	up	let-7c-5p	Oncogenic functions	Therapeutic target	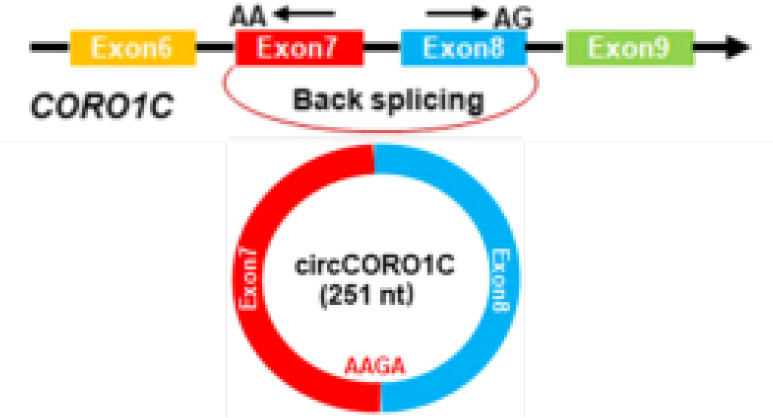	(108)
circCRIM1	chr2	CRIM1	EIciRNAs	NPC	up	–	Promoted metastasis and EMT	Prognosis	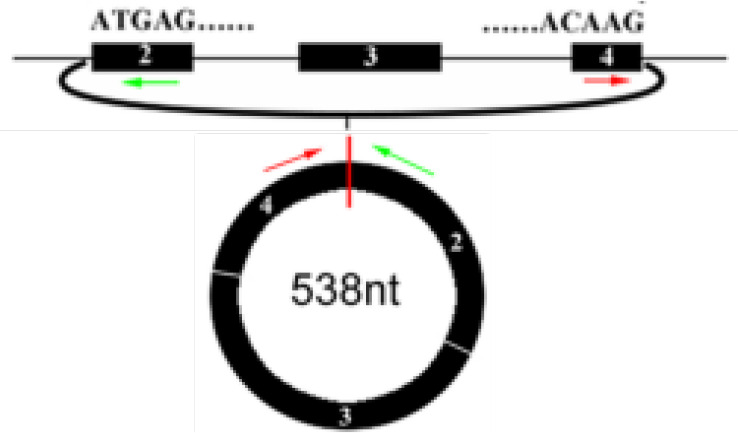	(109)
circ_0000140	chr1	KIAA0907	EIciRNAs	OSCC	down	miR-31, LATS2	Tumor suppressor	Therapeutic target	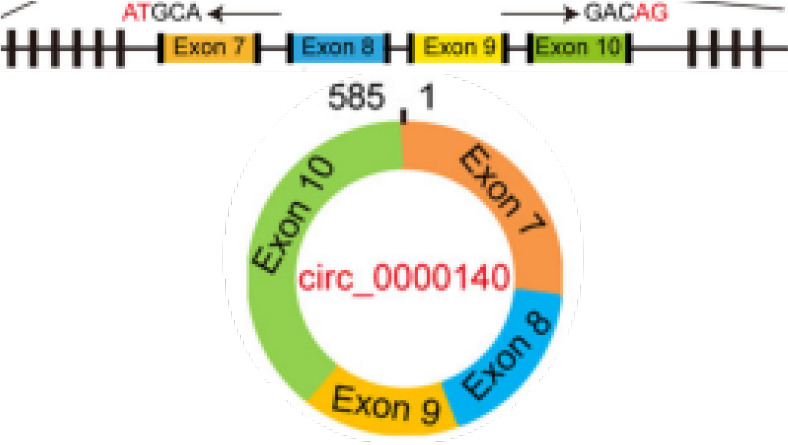	(110)
hg19_circ_0005033	–	–	–	LSCC	up	miR-4521	Oncogenic functions	Therapeutic target and biomarker	–	(119)
circ_0109291	chr19	ZNF714	EIciRNAs	OSCC	up	miR-188-3p	Promotes cisplatin resistance	–	–	(176, 177, 186)
circGNG7	chr19	GNG7	EIciRNAs	HNSCC	down	Ser78, Ser82	Tumor suppressor	Prognostic and therapeutic target	–	(133)
circ-PKD2	chr4	PKD2	EIciRNAs	OSCC	down	miR-646, Atg13	Increase cisplatin sensitivity	Prognosis	–	(134)
circCUX1	chr7	CUX1	EIciRNAs	HPSCC	up	caspase 1	Radiotherapy tolerance	Therapeutic target	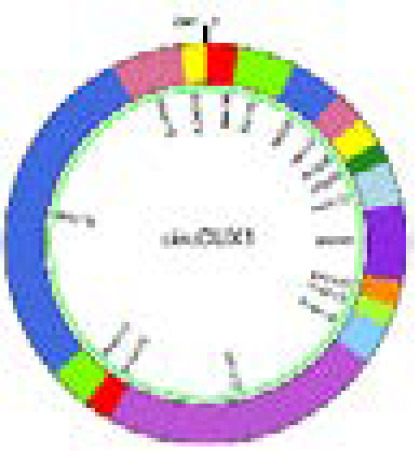	(135)
circBART2.2	–	–	–	NPC	up	IRF3	Promotes immune escape	Therapeutic target	–	(187)
circFAT1	chr4	FAT1	EIciRNAs	SCC	up	STAT3	Immunosuppressive environment	Therapeutic target	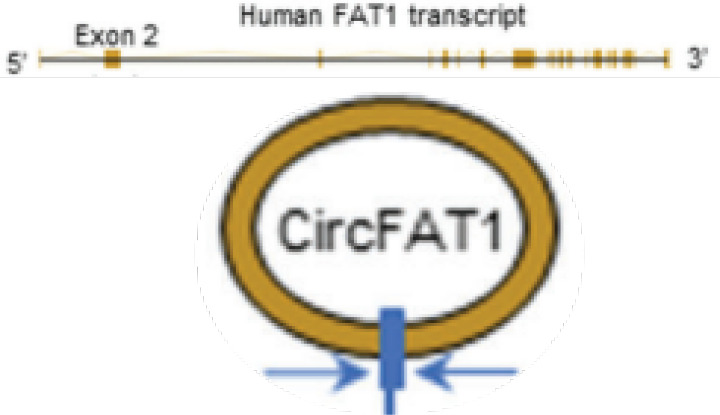	(122)

CircRNA, circular RNA; OSCC, oral squamous cell carcinoma; LSCC, laryngeal squamous cell carcinoma; NPC, nasopharyngeal cancers; HNSCC, head and neck squamous cell carcinoma; SCC, squamous cell carcinoma; HNSCC, head and neck squamous cell carcinoma.

C-Myc, a proto-oncogene and is highly expressed as a biomarker in different types of cancer (65), is also an important regulator for tumor cell proliferation. It has been shown that the circRNA circ-NOTCH1 plays an important regulatory role in the cell proliferation process of nasopharyngeal carcinoma (NPC) by targeting the miR-34c-5p/c-Myc axis (66) (**Figure 2** and **Table 1**). Alternatively, circCAMSAP1 facilitates the expression of SERPINH1, a molecular chaperone closely associated with tumor proliferation and metastasis, by improving its mRNA stability through the 3’-untranslated region (3’UTR). High-level expression of SERPINH1 can reduce the ubiquitination-mediated degradation rate of c-Myc, boosting cell proliferation and promotion of the occurrence of NPC (67) (**Table 1**). Wei Zhao et al. suggested that circUHRF1 acts as a foam of mir-526b-5p in OSCC, thereby positively regulating c-Myc, promoting cell proliferation and tumorigenesis in OSCC (68) (**Table 1**).

Nevertheless, tumor cells can evade from the supervision of inhibitory growth signals by blocking the expression or activation of tumor suppressors. MiR-7 is a tumor suppressor that regulates a variety of biological processes. In LSCC, the expression of CDR1as is negatively correlated with that of miR-7. Induced overexpression of CDR1as can promot the proliferation of two LSCC cell lines (Hep2 and AMC-HN-8). These effects, however, can be eliminated by knocking down CDR1as or overexpressing miR-7. This suggests that CDR1as acts as the miR-7 sponges. Further *in vivo* experiments showed that overexpression of CDR1as can upregulat Cyclin E1 (CCNE1) and PIK3CD, the key targets of miR-7, in Hep2 and AMC-HN-8 cells, increasing tumor proliferation index and facilitating tumor growth (25) (**Figure 2** and **Table 1**).

### CircRNAs regulate tumor cell cycle

Multiple anti-proliferative signals serve to maintain cellular quiescence in normal cells (54) by blocking cell proliferation in the manner of cell cycle arrest (69). Phosphatase and tensin homolog gene (PTEN), is a tumor suppressor that induce cell cycle arrest, mostly at G1phase in tumor cells (70). While the germline mutations in PTEN substantially increase the risk of cancer (71). CircANTRL1 may act as a sponge for miR-23a-3p to promote PTEN expression and induce cell cycle arrest, ultimately contributing to improvement of radiosensitivity in OSCC (72) (**Figure 2** and **Table 1**). Research also showed that silencing hsa_circ_0006232 in LSCC can enhance the expression of PTEN in tumor tissues and affect the process of LSCC (73) (**Table 1**).

In addition to regulation of anti-proliferative signals, circRNAs can regulate tumor growth by modulating cell cycle mediators. Cyclin D1, a cell cycle regulator protein encoded by CCND1, promotes the G1-S transition by activating cyclin dependent kinases 4 (CDK4) or cyclin dependent kinases 6 (CDK6). The expression level of circMYLK in LSCC carcinoma tissues was significantly higher than that in adjacent para-cancerous tissues, can increase the level of cell cycle protein D1 in Tu-177 cells (LSCC cell line) through miR-195/cyclin D1 axis, accelerate the G1-S transition of cells, and then promote the proliferation of tumor cells (74) (**Figure 2** and **Table 1**). Yan et al. showed that circ-CCND1 can increase CCND1 levels and promote LSCC cancer cell proliferation by interacting with HuR and miR-646 (75) (**Table 1**). Kuang et al. suggested that in OSCC, circ_0000745 promotes OSCC progression by acting as a miR-488 sponge and by interacting with HuR proteins to regulate the expression of CCND1 (76) (**Table 1**).

### CircRNAs are involved in resistance to tumor cell death

Apart from cell proliferation rate, cell death rate also determines the number of cells (54). Apoptosis and autophagy are the main mechanisms leading to cell depletion, and cancer cells have the ability to resist these mechanisms (69).

Tumor cells can develop resistance to apoptosis by several mechanisms. Mutations can result in the loss of pro-apoptotic regulators, most commonly in the p53 tumor suppressor gene (54). B-cell lymphoma 2 associated X-protein gene (Bax) is regulated by P53 gene and encodes B-cell lymphoma 2 (Bcl-2) homologous water-soluble protein BAX, which promotes cell apoptosis (77). Overexpression of Bax would antagonize the protective effect of Bcl-2 and lead to cell death. Bax can also regulate the expression of apoptotic protease activator 1 (Apaf-1) and affect the mitochondrial apoptosis pathway (78). Apaf-1 ultimately regulates the caspase-family related proteins such as caspase-3, the most important end cleaving enzyme in apoptosis (79). When hsa_circ_0055538 was overexpressed in OSCC cell lines SCC9 and CAL27 cells, the expression levels of p53, p21, Bax, Apaf-1, caspase-3, and cleaved caspase-3 are increased, while that of Bcl-2 is decreased. When hsa_circ_0055538 is adjusted downward, the opposite result as above is obtained, which suggests that hsa_circ_0055538 may regulate the apoptotic process of OSCC tumor cells through the p53 signaling pathway (80) (**Figure 2** and **Table 1**). Circ_0005320 knockdown can reduce the expression level of Bcl-2 and increase the expression level of BAX by regulating mir-637/mir-486-3p, thus promoting the apoptosis of OSCC cells (81) (**Table 1**).

Overexpression of Smad3 can promote apoptosis of many human cancers (82). Circ_0000218 was highly expressed in LSCC cells, while miR-139-3p was less expressed in LSCC cells and negatively regulated by circ_0000218. Silencing of circ_0000218 has been shown to suppress LSCC cell viability and promote apoptosis by negatively regulating miR-139-3p expression levels, while the effects can be reversed by Smad family member 3 (Smad3) overexpression. Thus, circ_0000218 inhibition inhibits the growth of LSCC by targeting the miR-139-3p/Smad3 axis (83) (**Figure 2** and **Table 1**).

Autophagy is a lysosome-dependent cellular degradation program that maintains energy metabolic homeostasis by eliminating toxic and potentially compromised cellular components, providing a source of nutrition and energy for cell survival in a starved state (84). Higher bioenergetic and nutritional requirements of cancer cells than normal cells. In the advanced process of malignancy, they may enable themselves to survive under low nutrient and hypoxic conditions by inducing autophagy (85). The signaling pathways involved in autophagy, including mammalian target of rapamycin (mTOR), phosphatidylinositol-3 kinase (PI3K)-protein kinase B (AKT) and mitogen-activated protein kinases (MAPK)/extracellular signal-regulated kinase (ERK), and protein kinase C iota type 1 (PRKC1) have been well established (86–88). Among them, PRKCI is a member of the kinase C protein family and is considered to be an important target for cancer therapy (89–91). It has been shown that PRKCI inhibits autophagy and promotes LSCC cell proliferation, migration, invasion, and chemoresistance. Qu et al. found that a decreasing expression of PRKCI in the U2OS cells (LSCC cell line) can enhance autophagy while suppress cell phenotype (92). In contrast, circPARD3 was able to inhibit Akt and mTOR phosphorylation and suppress autophagy in LSCC cells by activating the PRKCI-Akt-mTOR pathway, promoting malignant progression and chemoresistance in LSCC cells (93) (**Figure 2** and **Table 1**). In OSCC, circCDR1as was significantly increased in OSCC cancer tissues (94). Transcription factor EB (TFEB), a major regulator of lysosomal and autophagic function, is enhanced under hypoxic conditions, and it can coordinate autophagosomal degradation by driving autophagy and lysosomal gene expression (95). The expression level of lysosomal-associated membrane protein 2 (LAMP2) also steadily increases under hypoxic conditions. CircCDR1as can act as a sponge for miR-671-5p to promote autophagy in cells under hypoxic conditions, thereby inducing elevated levels of TFEB and LAMP2 and regulating lysosomal function. CircCDR1as also promotes hypoxia-induced AKT and inhibits mTOR activity, suggesting that AKT/mTOR pathway and lysosomal activity contribute to circCDR1as-induced activation of autophagy under hypoxic conditions in OSCC (96) (**Table 1**).

### CircRNAs regulate persistent tumor angiogenesis

The growth of tumors is often accompanied by angiogenesis. Vascular endothelial growth factor-A (VEGFA; also known as VEGF) is one of the main factors driving the generation and expansion of tumor vascular beds (97). Carbonic anhydrase 9 (CAIX) is involved in cancer angiogenesis under hypoxia (98). Tumor cells produce carbon dioxide intracellularly, which enters the extracellular environment by diffusion, resulting in a lower extracellular pH. Extracellular carbon dioxide undergoes a reversible hydration reaction in the presence of CAIX to produce bicarbonate and release protons. Subsequently, bicarbonate is transported into the cell by the Bt transporter protein, which binds to intracellular protons to form H_2_O and CO_2_. To overcome the lack of nutrients in tumor cells undera hypoxic state, vascular endothelial growth factor A (VEGFA) promotes angiogenesis by participating in the activation of intracellular pathways associated with angiogenesis (99), and for tumor cells, angiogenesis is an important process that promotes their metastasis (100). A recent study showed that hsa_circ_0001766 is involved in the ceRNA machinery in OSCC and plays an important role in OSCC cell progression through the hsa_circ_0001766/miR-877-3p/VEGFA axis (101) (**Figure 2** and **Table 1**).

Activation of the janus kinase-2 (JAK2)/activator of transcription 3 (STAT3) pathway has been reported to play a key role in a variety of oncogenic processes, including angiogenesis. In OSCC, circ_0005320/miR-486-3p or circ_0005320/miR-637 axis can activate the JAK2/STAT3 pathway on OSCC cells and promote angiogenesis (81).

### CircRNAs regulate tumor invasion and metastasis

It is reported that 90% of death in cancer patients are caused by metastases rather than the primary tumor (102). During the progression of most cancers, tumor cells can migrate from the primary site, forming new colonies at distant sites. Epithelial-mesenchymal transition (EMT) has been shown to be an essential process in carcinoma cell migration and tissue metastasis (103). EMT involves a process of cellular reprogramming that convert epithelial cells into a mesenchymal-like phenotype. And this process is characterized by the loss of epithelial surface markers such as E-cadherin and the acquisition of mesenchymal markers such as vimentin and N-cadherin (104, 105). It has been demonstrated that circRPMS1 can act as a sponge for miR-203, miR-31, and miR-451 to promote the deletion of E-cadherin and the upregulation of N-cadherin and vimentin in the NPC cell line C666-1 cells, thereby regulating the EMT and invasiveness of NPC cells (106) (**Figure 2** and **Table 1**). In OSCC, circEPSTI1 functions as a sponge for miR-942-5p to activate EMT, which in turn promotes migration and invasion of OSCC cells (107) (**Table 1**). In addition, circCORO1C can bind to let-7c-5p and block it to reduce the level of Pre-B-cell leukemia homeobox transcription factor 3 (PBX3), thus promoting EMT and stimulating LSCC cell invasion and migration *in vitro* and *in vivo* (108) (**Table 1**). CircCRIM1 is overexpressed in NPC cells and distantly metastasized NPC tissues, and it induces EMT during the progression of NPC. When circCRIM1 expression was knocked down in NPC cell lines S18 and HONE1, NPC cell morphology changed from a spindle or elongated mesenchymal morphology to an epithelial morphology. Meanwhile, the expression of the epithelial marker E-cadherin was significantly increased and the expression of the mesenchymal markers N-cadherin and Vimentin were decreased in NPC cells, suggesting that circCRIM1 has oncogenic potential and may be a marker for predicting NPC metastasis (109) (**Table 1**). Circ_0000140 derived from exons 7-10 of the KIAA0907 gene is downregulated in OSCC tissues and negatively correlates with the prognosis of OSCC patients. Further studies have shown that circ_0000140 can bind to miR-31 and upregulate its target gene large tumor suppressor kinase 2 (LATS2), thereby affecting the EMT of OSCC cells (110) (**Table 1**).

Epithelial splicing regulatory protein 1 (ESRP1), also known as RNA-binding motif protein 35A (RBM35A), is a key component in the EMT process in malignant tumors (111–114), and it has been shown to be one of the splicing factors associated with EMT in tumor metastasis (8). The circUHRF1/miR-526b-5p/c-Myc axis was found to promote ESRP1 transcript levels in OSCC, and ESRP1 was also identified to target the flanking introns of circUHRF1 and accelerate its cyclization, forming a circUHRF1/miR-526b-5p/c-Myc/TGF-β1/ESRP1 feedback loop in the EMT progression (68) (**Figure 2** and **Table 1**).

### CircRNAs regulate the stemness of HNSCC

Cancer stem cells (CSCs) are a fraction of cancer cells that have the ability to renew and initiate themselves, and are responsible for the metastasis and spread of cancer cells in the body and the failure of treatment (115–117). CircRNAs have been shown to regulate the activity of tumor stem cells. CD133^+^ and CD44^+^ CSCs, also known as TDP cells, isolated from LSCC cells and highly express the stem cell markers Sex determining Region Y (SOX2) and octamer-binding transcription factor 4 (OCT4), have enhanced proliferation, migration, and colony formation, and are more resistant to chemotherapy and radiotherapy (118). Compared with parental cells, RNA sequencing of TDP cells showed 3684 differentially expressed circRNAs (*p* < 0.01, log2FC > 1) (119). Among them, Hg19_circ _0005033 upregulated in TDP cells, and acted as a sponge for miR-4521 to upregulate the target STAT5A expression thus inducing stem cell-like cells (120). Therefore, Hg19_circ_0005033 may support the stem cell properties of CD133^+^ CD44^+^ laryngeal CSCs through miR4521/STAT5A axis, which needs to be verified by further studies. SOX2 has been shown to regulate self-renewal and tumorigenicity of stem cell-like cells in HNSCC (121) (**Figure 2** and **Table 1**). In nude mouse models with HNSCC, circFat1 KD can significantly inhibit SOX2 + cells and tumor stemness (122).

### CircRNAs regulate chemotherapy and radiation therapy resistance in tumors

For the treatment of locally advanced disease, radiation therapy is used as an adjunct to surgery or in conjunction with chemotherapy (2). However, acquired chemotherapy resistance is one of the main reasons for treatment failure in patients with advanced tumors (123). ATP-binding cassette B1 (ABCB1) is a multidrug resistance-associated protein that is highly expressed in resistant cell lines and can promote chemotherapy resistance by pumping intracellular drugs extracellularly (124, 125). Therefore, inhibition of ABCB1 expression is an effective way to reduce tumor drug resistance (126). Circ_0109291 promotes cisplatin resistance in oral squamous carcinoma by regulating ABCB1 expression mainly through adsorption of miR-188-3p. This provides a theoretical basis for reducing the incidence of drug resistance in OSCC (127) (**Figure 2** and **Table 1**). Heat shock protein 27 (HSP27, also known as HSPB1), is a member of the small heat shock protein superfamily with Phospho-HSP27 (Ser15), Phospho-HSP27 (Ser78), and Phospho-HSP27 (Ser82) receptor sites (128). The function of HSP27 is regulated by post-translational phosphorylation (129, 130). HSP27 enhances multidrug resistance in tongue squamous cell carcinoma (SCCT) by activating NF-κB (131). In LSCC, HSP27 overexpression creates cellular resistance to various cellular agents, such as cisplatin and staurosporin, by inducing cell cycle arrest and remodeling actin polymerization associated with drug uptake, respectively (132). CircGNG7 can block the phosphorylation sites of Ser78 and Ser82 and inhibit the phosphorylation of HSP27 in HNSCC, thus inhibiting the phosphorylation of HSP27 in the malignant signaling cascade, which may therefore reduce the chemotherapeutic resistance of HNSCC (133) (**Table 1**). In addition, in OSCC, circ-PKD2 is a tumor suppressor gene that promotes autophagy related 13 (Atg13) expression by sponging miR-646, thereby accelerating cisplatin sensitivity (134) (**Table 1**).

Radiotherapy tolerance is also an important prognostic predictor for patients with HNSCC (135). CircCUX1, upregulated in patients with radiation-resistant hypopharyngeal squamous cell carcinoma (HPSCC), predicts poor survival outcomes. CircCUX1 binds to caspase 1 (a member of the caspase aspartate-specific protease family, also known as interleukin-1 β invertase) (136) mRNA and inhibits its expression, thus modulating the inflammatory response of tumor cells to radiation therapy and thereby producing radiation therapy tolerance (135) (**Figure 2** and **Table 1**).

### CircRNAs regulate immune escape

The programmed cell death-1/programmed cell death ligand-1 (PD-1/PD-L1) signaling pathway is an important mechanism mediating tumor immunosuppression (137, 138). PD-L1 is often expressed on the surface of tumor cells and immunosuppressive cells, and interacts with PD-1 on T cells, thus preventing tumor antigen-specific T cells from activating and killing tumor cells and leading to tumor immune escape (139–144). It has been shown that circBART2.2, which is highly expressed in NPC, can activate the transcription factors interferon regulatory Factor 3 (IRF3) and nuclear factor kappa-B (NF-κB) upon binding to the decapping enzyme region of retinoic acid inducible gene protein I (RIG-I), which promotes the transcription of PD-L1 and thus leads to tumor immune escape (122) (**Figure 2** and **Table 1**).

Juan Sun et al. proposed the following hypothesis: hsa_circ_001859, hsa-circ_001373, and hsa_circ_002179 may regulate the expression of PD-L1 and immunosuppressive molecule IL-10 through the ceRNA network to affect the immune evasion of LSCC, and then affect the degree of malignancy of LSCC. This hypothesis is likely to represent an important mechanism of immune evasion in LSCC, however, this hypothesis needs to be tested by large-scale scientific studies in the future (145).

### CircRNAs regulate tumor microenvironment

Since 1989, the association between cancer and the tumor microenvironment (TME) has received increasing attention with the “seed and soil theory” hypothesis proposed by Stephen Paget (146, 147). Growing amount of evidences have confirmed that tumor cells must recruit and reprogram surrounding normal cells in order to promote tumor progression (148). TME is mainly composed mainly of stromal cells and extracellular matrix (ECM) components. Stromal cells include immune cells, cancer-associated fibroblasts (CAFs), endothelial cells, and pericytes.

ECM is a highly dynamic structural network consisting of many stromal components bearing continuous remodeling mediated by several stromal degrading enzymes during tumorigenesis and progression (149). The protease matrix metalloproteinases (MMP) family supports the tumor cell invasion toward the basement membrane and stroma, the penetration of blood vessels, and metastasis by interacting with macromolecules on the basement membrane to degrade and stimulate ECM remodeling (150). Silencing of circZNF609 inhibited the proliferation and invasiveness of LSCC cells, and significantly increased the expression of MMP-2 protein (64), which may affect the degradation and remodeling of ECM in LSCC (**Table 1**). Moreover, in other tumors, like gastric cancer cells, protein expression levels of VEGF and migration-associated proteins MMP-2 and MMP-9 were significantly reduced after knockdown of circ-0000096, suggesting that circ-0000096 may affect cell growth and migration by regulating stromal remodeling and angiogenesis (151), as well as the TME. CircLMNB1, which is highly expressed in colorectal cancer (CRC), can downregulate MMP-2 and MMP-9 expression and inhibit EMT when its expression is knocked down, thereby affecting tumor dissemination and invasion (152). Silencing circDENND4C under hypoxic conditions downregulates the protein expression levels of MMP-2 and MMP-9 (153). All these provide a basis for future studies on the changes of TME in HNSCC.

Growing evidence suggests that the innate immune cells (macrophages, neutrophils, dendritic cells, innate lymphocytes, myeloid suppressor cells, and NK cells) as well as adaptive immune cells (T cells and B cells) contribute to tumor progression when present in TME (154). HNSCC is well known for having an immunosuppressive TME with low number of tumour infiltrating lymphocytes (155). In particular, the infiltration of CD8^+^ T cells into the TME was low. Increasing the expression of circFAT1 in HNSCC can significantly increase the infiltration of CD8^+^ T cells into tumors, which is an important molecular mechanism mediating the immunosuppressive environment of HNSCC (122) (**Figure 2** and **Table 1**).

CircRNAs can interact with stromal cells, fibroblasts, and endothelial immune-related molecules in the TME of a variety of tumors, as well as various immune cells such as macrophages (156). However, the key role of more circRNAs in the TME of HNSCC is a challenge to be faced in the future.

## Discussion

There are growing evidences that circRNAs have great potential in the diagnosis, treatment and prognosis of HNSCC. It is worth mentioning that regulatory effects of circRNAs described in our review are not independent of each other, but is usually closely related to various biological behaviors of cancer cells. For example, circZDBF2 promotes malignant cell behavior in OSCC, including cell proliferation, invasion, migration, and EMT processes (157). Upregulation of circRNA_100290 expression promotes proliferation, migration and invasion of LSCC cells while inhibiting apoptosis (158).

Current studies mostly focus on the clarification of mechanism of circRNAs based on full transcription sequencing of cancer tissues and normal adjacent tissues, and lack of circRNA expression data at the single-cell level of HNSCC. And these studies may be crucial for our future insight into circRNA function and advance the development of new biomarkers. However, to date, only a small number of functional circRNAs identified in HNSCC. Most of these circRNAs regulate the progression of HNSCC through miRNA sponge function. In addition, circRNAs interact with functional proteins, some of which have protein-coding potential. For example, circGNG7 mentioned in this paper can bind to serine residues 78 and 82 of HSP27 to reduce carcinogenic signal transduction.

Recent studies have also shown that circRNAs can regulate immune metabolism of HNSCC, and circ_0008068 may affect glycolysis by targeting mir-153-3p/acylglycerol kinase (AGK) axis and facilitate the CD8^+^ T cell response (159). It provides a better understanding of the interaction between HNSCC cellular immunity and metabolic rate, and we look forward to opening up new immune-related therapies for HNSCC. The potential network of circRNAs-RBPs and other functions of circRNAs in HNSCC have important research value and potential application value. It is expected that circRNAs can be used as important biomarker for clinical diagnosis and treatment of HNSCC.

Studies have shown that circRNA has great potential in diagnosing pathological types of lung cancer (160) and molecular subtypes of breast cancer (161). Moreover, hsa_circ_0000190 and 0001649 could be used as biomarkers to predict recurrence and treatment response in patients with OSCC (162). CircRNAs have the advantages of high abundance and good stability, and can be detected in body fluids and blood (163, 164), so it can be used as ideal diagnostic and predictive biomarkers for HNSCC diagnosis. In addition, Zhuo et al. analyzed the diagnostic value of various RNAs in hepatocellular carcinoma and with result showing that circRNAs > lncRNAs > microRNAs (165).

CircRNAs have shown great potential as therapeutic molecular tools in many cancers such as HNSCC (110, 166, 167), breast cancer (168), hepatocellular carcinoma (169), papillary thyroid cancer (170) gastric cancer (171). Compared with existing linear miRNA antagonists, circRNAs are structurally more stable, more resistant to degradation by nucleases without chemical modification, and well tolerated *in vivo* (172). The knockdown of oncogenic circRNAs in preclinical animal models validated circRNAs RNAi effect on tumor treatment (169, 173, 174). Some researchers have attempted to design and synthesize tumor suppressive circRNAs *in vitro* for delivery into disease models to explore the potential of circRNA-like drug development. In the treatment of heart disease (172) and gastric malignancies (175), engineered circRNAs were delivered to *in-vivo* and *in-vitro* disease models to reverse pathogenic mechanisms. CircRNAs will be better diagnosis and treatment targets for HNSCC patients, which will benefit for the translation of basic to clinical medicine.

In conclusion, although the technical challenges concerning circRNAs have not been fully overcome yet, with the development of biological methods, informatics techniques, and further research, we will gain more and more understanding of the physiological functions and roles of circRNAs. The new diagnostic and therapeutic strategies for HNSCC based on circRNAs will effectively serve for the clinical work in the future.

## Author contributions

XH and RT conceived the paper and drafted the manuscript. XH and CW drew the figures. XS and YL edited and finalized the manuscript. All authors contributed to the article and approved the submitted version.

## Funding

This work was supported by Taishan Scholars Project (No. ts20190991).

## Conflict of interest

The authors declare that the research was conducted in the absence of any commercial or financial relationships that could be construed as a potential conflict of interest.

## Publisher’s note

All claims expressed in this article are solely those of the authors and do not necessarily represent those of their affiliated organizations, or those of the publisher, the editors and the reviewers. Any product that may be evaluated in this article, or claim that may be made by its manufacturer, is not guaranteed or endorsed by the publisher.
